# UV‐Triggered Hydrogel Coating of the Double Network Polyelectrolytes for Enhanced Endothelialization

**DOI:** 10.1002/advs.202401301

**Published:** 2024-03-28

**Authors:** Xing‐wang Wang, Yi‐jing Yin, Jing Wang, Hong‐mei Yu, Qian Tang, Zhao‐yang Chen, Guo‐sheng Fu, Ke‐feng Ren, Jian Ji, Lu Yu

**Affiliations:** ^1^ Key Laboratory of Cardiovascular Intervention and Regenerative Medicine of Zhejiang Province, Department of Cardiology, Sir Run Run Shaw Hospital Zhejiang University School of Medicine Hangzhou 310016 China; ^2^ MOE Key Laboratory of Macromolecular Synthesis and Functionalization, Department of Polymer Science and Engineering Zhejiang University Hangzhou 310058 China; ^3^ Department of Surgery, Sir Run Run Shaw Hospital Zhejiang University School of Medicine Hangzhou 310016 China; ^4^ Engineering Research Center for Cardiovascular Innovative Devices of Zhejiang Province Hangzhou 310016 China

**Keywords:** device‐related thrombus, double network polyelectrolyte hydrogel coating, endothelialization, left atrial appendage occluder, UV‐triggered polymerization

## Abstract

The left atrial appendage (LAA) occluder is an important medical device for closing the LAA and preventing stroke. The device‐related thrombus (DRT) prevents the implantation of the occluder in exerting the desired therapeutic effect, which is primarily caused by the delayed endothelialization of the occluder. Functional coatings are an effective strategy for accelerating the endothelialization of occluders. However, the occluder surface area is particularly large and structurally complex, and the device is subjected to a large shear friction in the sheath during implantation, which poses a significant challenge to the coating. Herein, a hydrogel coating by the in situ UV‐triggered polymerization of double‐network polyelectrolytes is reported. The findings reveal that the double network and electrostatic interactions between the networks resulted in excellent mechanical properties of the hydrogel coating. The sulfonate and Arg‐Gly‐Asp (RGD) groups in the coating promoted hemocompatibility and endothelial growth of the occluder, respectively. The coating significantly accelerated the endothelialization of the LAA occluder in a canine model is further demonstrated. This study has potential clinical benefits in reducing both the incidence of DRT and the postoperative anticoagulant course for LAA closure.

## Graphical abstract

In this study, a hydrogel coating of double‐network polyelectrolytes on the cover disk of the left atrial appendage occluder is designed. The hydrogel coating binds stably to the occluder and promotes hemocompatibility and endothelial growth on the occluder. The hydrogel coating enhances endothelialization of the occluder in canine vivo, which has potential clinical benefits.

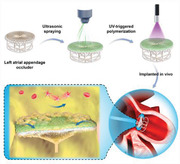

## 1 Introduction

Atrial fibrillation (AF) is a common arrhythmia affecting 2%–4% adult population worldwide.^[^ ^1^ ^]^ ≈25% of ischemic strokes are attributed to atrial fibrillation, many of which are due to a cardiogenic embolism originating from the left atrial appendage (LAA).^[^ ^2^ ^]^ Percutaneous closure of the LAA with occluders has emerged as a common preventive option for patients with AF who are at risk of stroke, as it can significantly reduce the incidence of stroke and systemic embolism.^[^ ^3^ ^]^ Commercially available LAA occluders are typically composed of a super‐elastic metal (usually a nickel‐titanium alloy, NiTi alloy) that is capable of fixating on the LAA, as well as a polymer fabric (usually polyethylene terephthalate, PET) that is capable of impeding abnormal blood flow.^[^ ^1^ ^]^ However, the poor biocompatibility of these materials may lead to delayed endothelialization and thrombogenesis on the surface,^[^ ^4^ ^]^ resulting in device‐related thrombus (DRT).^[^ ^5^ ^]^ The risk of DRT accumulates continuously when the device comes into long‐term contact with blood. DRT occurs in 4%–7% of patients and is associated with higher rates of ischemic stroke and systemic embolism.^[^ ^5^, ^6^ ^]^ Therefore, addressing the issue of DRT when using LAA occluders for stroke prevention in patients with AF is crucial.

Designing coatings for occluders that provide anticoagulant properties and promote endothelialization without altering the constituent material is an important strategy for addressing DRT.^[^ ^7^ ^]^ Polyelectrolyte hydrogel coatings exhibit excellent biocompatibility and can immobilize multiple functional groups on the coating surface via covalent interactions.^[^ ^8^ ^]^ As a result, they are gradually emerging as an important technology for medical coatings. Polyelectrolytes containing sulfonic acid groups have demonstrated excellent blood and cell compatibility both in vitro and in vivo.^[^ ^9^ ^]^ Additionally, the Arg‐Gly‐Asp (RGD) peptide, a cell‐specific adhesion polypeptide, has been conjugated to polyelectrolytes to provide a matrix for numerous types of cell adhesion and migration.^[^ ^10^ ^]^ Immobilizing sulfonic acid groups and RGD peptides within the polyelectrolyte hydrogel coating is promising for providing anticoagulant and rapid endothelialization capabilities to occluders.

Notably,occluders have complex structures, large surface areas, and multivariate morphologies. Owing to the requirement of precise positioning, occluders undergo repeated friction with the sheath during deployment before fully closing the LAA. This indicated that the coating exhibited superior mechanical properties. The design of hydrogels with strong physical interactions effectively enhances their toughness. Gong et al. reported a physical hydrogel with oppositely charged polyelectrolytes ^[^ ^11^ ^]^ and found that it had strong viscoelasticity and high toughness. We recently reported a polyelectrolyte hydrogel coating prepared via in situ photopolymerization.^[^ ^12^ ^]^ The coating exhibited favorable toughness and stable adhesion to various substrates via electrostatic interactions. Moreover, in‐situ photopolymerization expedites the coating preparation process.^[^ ^8^, ^13^ ^]^

**Scheme 1:**
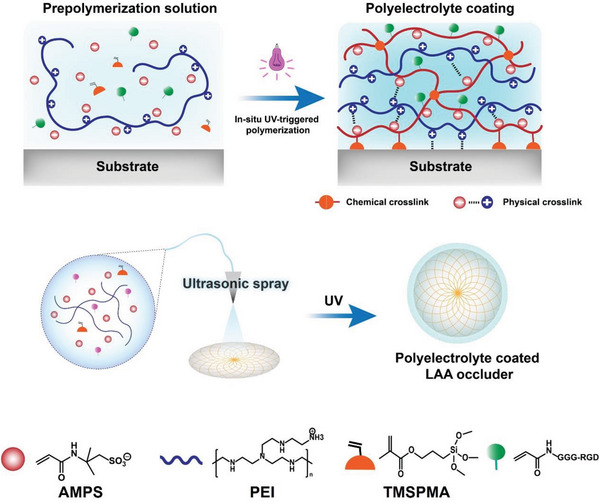
Schematic illustration of the polyelectrolyte coating on LAA occluder.

In this study, we prepared a double‐network polyelectrolyte hydrogel coated with sulfonic acid groups and an RGD peptide on the surface of an LAA occluder using in situ ultraviolet‐triggered (UV‐triggered) polymerization. As illustrated in **Scheme** **1**, the coating was composed of two polyelectrolytes, polyethylenimine (PEI) and poly(2‐acrylamido‐2‐methyl‐1‐propanesulfonic acid) (PAMPS). The sulfonic acid groups of PAMPS not only provide good hemocompatibility to the coating but also enhance the mechanical properties of the double network hydrogel by electrostatic interactions with the PEI amino group. Polycation PEI endows the coating with ultra‐tough binding through a polycation‐reinforced surface‐binding strategy^[^ ^14^ ^]^ and wettability for spray coating. The silane coupling agent in the coating formed a covalent network and bonded to the hydrogel‐coating interface. RGD promotes rapid endothelialization of occluders by promoting cell adhesion and migration. We characterized the physical and anticoagulant properties of the coating and examined the behavior of endothelial cells (ECs) on the coating via in vitro cell culture and RNA sequencing. The genes involved in endothelialization were clustered and analyzed. We further verified the accelerated endothelialization of the occluder using a canine LAA model.

## 2 Results and Discussion

### 2.1 Preparation and Characterization of the PEI/PAMPS Coating on PET

To achieve durable and stable adhesion of the coating to the LAA occluder, we employed a combination of electrostatic forces and chemical anchoring to achieve a stable hydrogel coating on the substrate. The precursor solution for the hydrogel coating contained four components: 1) polycation PEI, which afforded the gel precursors with adjustable viscosities and enhanced wettability, making them suitable for spray coating; 2) anionic monomer AMPS (subsequently polymerized into polyanion PAMPS), capable of forming a stable hydrogel through electrostatic interaction with PEI and providing anticoagulant ability; 3) silane coupling agent 3‐(trimethoxysilyl) propyl methacrylate (TMSPMA), capable of forming chemical bonds with plasma‐treated surfaces; 4) cell adhesion peptide RGD, enhancing the cell compatibility of the coating. The combination of industrial coating techniques (spray coating) and in situ UV polymerization significantly accelerated the preparation of the hydrogel coating. For the LAA occluder with a diameter of 30 mm, the precursor solution was sprayed over the entire occlusion surface within 10 min, and the coating was fully polymerized after 5 min of UV irradiation (365 nm) (Scheme 1).

The charge balance condition was maintained to optimize the stability of the coating. First, we investigated the effect of the molar ratios of AMPS and PEI in the precursor solution on the coating. We chose five formulas that had PAMPS (mole %): PEI (mole %) of 35:65 (A35), 40:60 (A40), 45:55 (A45), 50:50 (A50), and 55:65 (A55) (Table S1, Supporting Information). The final composition of the coating was confirmed via elemental analysis (Table S1, Supporting Information). The results showed that with an increase in the AMPS ratio in the coating solution, the swelling ratio of the hydrogel coating increased (Table S1, Supporting Information) and the coating thickness increased. At higher PEI content (A35), the coating was partially dissolved (Figure S1, Supporting Information). At higher APMS contents (A50 and A55), the coatings exhibited severe swelling (Figure S1, Supporting Information). The zeta potential and water contact angle of the coating decreased with increasing AMPS content. These results indicated that a higher AMPS ratio increased the hydrophilicity of the coating, but excessive AMPS led to an unstable coating. The A45 coating exhibited a favorable swelling ratio and hydrophilicity, and its zeta potential was close to zero, indicating a balanced charge in the hydrogel coating. Therefore, A45 was selected as the optimal formula for further research.

**Figure 1:**
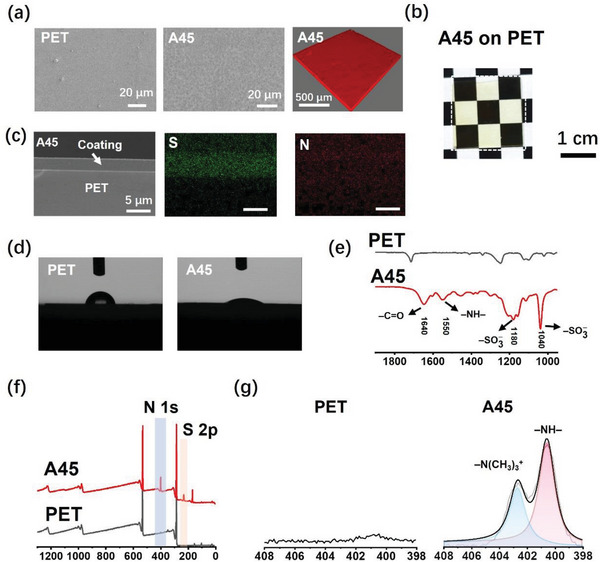
Characterization of PEI/PAMPS coating. a) SEM images of the surface of PET sheet and A45 coated PET and confocal scanning image of the A45 coating on the PET sheet. The coating was dyed using rhodamine B for confocal scanning. Scale bars, 20 µm in SEM images, and 500 µm in confocal scanning images. b) Photograph of A45 coating on the PET. Scale bar, 1 cm c) Cross‐section of SEM and elemental mapping image of the A45 coating. Scale bar, 5 µm. d) Photographs of the water contact angle on the PET and A45 coating. FT‐IR spectrum, e) XPS results, f) and high‐resolution spectrum of N1s g) of the PET and A45 coatings.

The surface topography of A45 coating was investigated (**Figure** **1**a,b). The prepared coating was flat, non‐porous, and transparent to the PET surface. The thickness of A45 coating was ≈3 µm (Figure 1c). The chemical composition of A45 coating was investigated via FTIR, EDAX, and XPS (Figure 1c–g). The results indicate that the coating was successfully formed and produced a hydrophilic surface (Figure 1d) on the PET substrate. S and N were uniformly distributed within the A45 coating (Figure 1c), suggesting a uniform distribution of PAMPS and PEI. We further confirmed the chemical composition of the A45 coating using FT‐IR spectroscopy, XPS, and elemental analysis. Compared to PET, N 1s and S 2p peaks were observed for the A45 coating (Figure 1f). The N 1s high‐resolution scan results (Figure 1g) and elemental analysis further demonstrated that the ratio of PAMPS to PEI in the hydrogel coating was consistent with our expectations (Table S1, Supporting Information). Based on these results, we concluded that the coating composition was precisely controlled, and the ratio of polyanions to polycations significantly affected the coating properties. Finally, we optimized the ratio to obtain a stable hydrogel coating with favorable properties, including a near‐neutral surface charge, low swelling ratio, and good hydrophilicity.

### 2.2 Hemocompatibility of the A45 Coating

**Figure 2:**
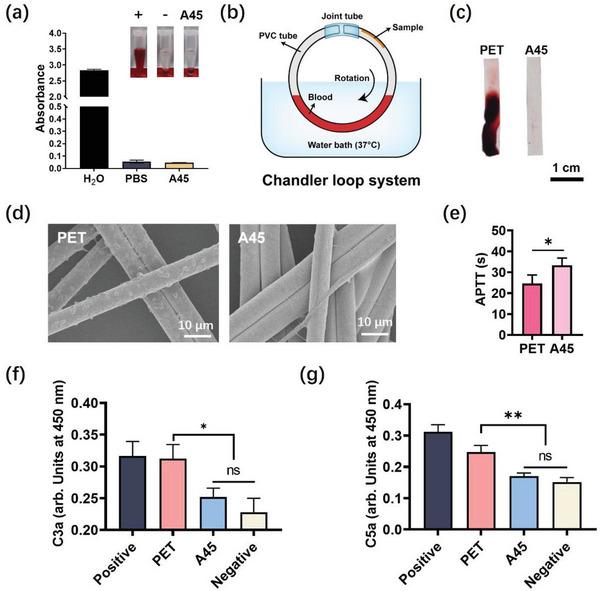
Blood compatibility of the A45 hydrogel coating. a) The absorbance at 540 nm of blood samples incubated with A45 coating. Saline and water were set as a negative and positive control, respectively. b) Illustration of the Chandler loop system. c) Photographs of the PET and A45 surface after 30 min of blood flow. Scale bar, 1 cm. d) SEM images of platelet adhesion on the PET fabric and A45 coated PET fabric. Scale bar, 10 µm. e) APTT of the poor platelet plasm on the PET surface and A45 coated surface. (f) C3a and g) C5a assays of the complement activation of the A45 coating. The data were representative of three independent experiments and expressed as the mean ± standard deviation (SD) (*n* = 3). Significance was determined using an unpaired t‐test, ^**^ *p* < 0.01, ^*^ *p* < 0.05, and ns were considered as not significant (*p* > 0.05).

We performed a series of experiments to evaluate the hemocompatibility of the A45 coating. We first investigated the hemolysis induced by the A45 coating. After incubation and centrifugation, the supernatant of the A45 coating was as colorless as that of the negative control (PBS), and the absorbance at 540 nm was not different from that of the negative control (**Figure** **2**a), which was considered safe for the blood‐contact coating. We further evaluated the anticoagulant behavior of the A45 coating in dynamic blood. The Chandler loop system simulated extracorporeal blood circulation (Figure 2b). After the loop, a large thrombus was observed on the PET surface, whereas no evident thrombus was observed on the A45 coating (Figure 2c). This result indicates that the hydrogel coating reduced the risk of thrombosis on the surface in a simulated internal blood flow environment. The longer activated partial thromboplastin time (APTT) on the A45 surface confirms the anticoagulant properties of the A45 coating. Countless platelets adhered to the PET fabric surface and showed evident pseudopodia and spreading characteristics (Figure 2d), whereas very few platelets adhered to the A45‐coated PET fabric (Figure 2d). We also observed a prolonged APTT on the A45 surface (Figure 2e). Activation of the complement system plays a critical role in coagulation.^[^ ^15^ ^]^ Complement fragment 3a (C3a) and complement fragment 5a (C5a) were chosen to evaluate complement activation after incubation with the A45 coating. The levels of C3a and C5a in the blood incubated on the A45‐coated surface were similar to those of the negative control, which were significantly lower than those of the blood incubated on PET (Figure 2f,g). These results indicated that the A45 coating had good hemocompatibility and prevented surface thrombus formation, possibly due to inhibition of the complement activation system. These results suggest that the A45 coating may exert inhibitory effects on inflammatory responses by inhibiting complement activation.^[^ ^9b^ ^]^

### 2.3 Evaluation of the Behavior of HUVECs on A45/RGD Coating

**Figure 3:**
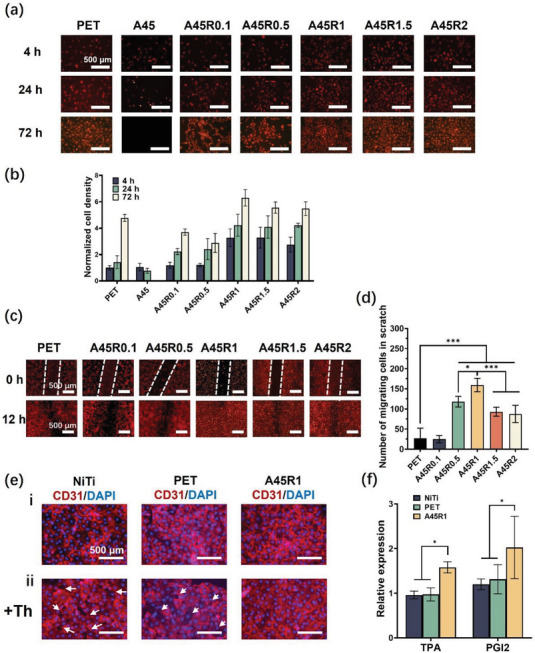
Effect of HUVECs behavior on A45/RGD coating. Fluorescence image a) and corresponding normalized cell density b) about HUVECs adhesion and proliferation on the PET and A45 coating with different RGD content at 4, 24, and 72 h. Scale bars, 500 µm. Fluorescence image c) and statistics on the number of migrating cells in 500 µm scratch d) on the PET and A45 coating with different RGD content at 0 and 12 h. Scale bars, 500 µm. e) Immunofluorescence images of endothelium morphology without thrombin stimulation i) and with thrombin (Th) stimulation ii) on the NiTi, PET, and A45R1. Blue fluorescence corresponds to cell nuclei stained with DAPI and red fluorescence corresponds to the CD31. Arrows indicate the gaps in the endothelium. f) The concentrations of tPA and PGI2 released in the culture media from HUVECs grown on the the NiTi, PET, and A45R1. The data were representative of three independent experiments (n = 3) and expressed as the mean ± SD. Significance determined using an unpaired t‐test, ^***^ *p* < 0.001, ^*^ *p* < 0.05, and ns were considered as not significant (*p* > 0.05).

Biomaterial surfaces have profound effects on cell behavior and ultimately influence the performance of medical devices. Rapid endothelialization of the occluder surface reduces the risk of long‐term complications. EC adhesion, proliferation, and migration are crucial for surface endothelialization, during which the concentration of the RGD peptide is critical.^[^ ^10a^ ^]^ We first optimized the RGD content of the A45 coating to achieve rapid cell migration and high cell adhesion. We used the AAc‐GGG‐RGD peptide in which one end of the RGD peptide was modified with a double bond (Figure S2, Supporting Information). We selected a series of RGD contents of 0.1% (A45R0.1), 0.5% (A45R0.5), 1% (A45R1), 1.5% (A45R1.5), and 2% (A45R2) in AMPS moles. As the RGD content gradually increased, cell adhesion (4 h) and proliferation (24 h, 72 h) increased. When the RGD content exceeded 1% (A45R1, A45R1.5, and A45R2), the adhesion and proliferation of the HUVECs on these coatings surpassed those on the PET surface (**Figure** **3**a,b). However, a further increase in RGD content (A45R1.5, A45R2) did not significantly increase cell adhesion and proliferation (Figure 3b). On the surface without RGD (A45), the initial HUVEC adhesion was minimal, and all adhered cells died after 72 h of culture (Figure 3a). We further examined the migration of cells on the A45 coatings with different RGD contents (Figure 3c). By counting the number of migrating cells in scratch assays, we found that when the RGD content exceeded 0.5% (A45R0.5), cell migration exceeded that on the PET surfaces (Figure 3d). When the RGD content reached 1% (A45R1), the migration speed reached its maximum, and increasing the RGD content (A45R1.5, A45R2) further decreased the cell migration speed (Figure 3d). These results showed that the A45R1 coating with better cell adhesion, proliferation, and migration was the most suitable coating for the LAA occluder.

### 2.4 Effects of the HUVEC Monolayer on A45R1 Coating

Good EC adhesion and rapid migration are advantageous for endothelial formation and maintenance.^[^ ^16^ ^]^ We further investigated the functionality and stability of the endothelial layer on the A45R1 surface. Immunofluorescence staining for CD31 in the endothelial layers on the PET, NiTi alloy, and A45R1 surfaces revealed that the expression of CD31 in the cells on the A45R1 coating was complete and continuous, and the HUVEC monolayer was compact (Figure 3e(i)). Thrombin is a protease produced by the damaged endothelium, capable of disrupting the barrier function of the endothelium.^[^ ^17^ ^]^ Thrombin is widely used to assess the endothelial layer.^[^ ^18^ ^]^ Following thrombin stimulation, the endothelial layer on the A45R1 surface remained intact without gaps or disruptions compared to the EC monolayers on the PET and NiTi alloy surfaces (Figure 3e(ii)). This revealed a tighter interaction between cells in the endothelial monolayer on the surface of the A45R1 coating; therefore, thrombin stimulation was not sufficient to induce separation between the cells. Tightly joined ECs within the endothelial monolayer can resist external stimuli and produce various substances with antithrombotic effects, which are beneficial for the long‐term inhibition of thrombosis. Therefore, we determined the secretion of antithrombotic molecules by endothelial cells on different substrates. The tissue plasminogen activator (tPA) released by healthy endothelial cells is a serine protease that plays a crucial role in fibrinolysis by converting plasminogen into plasmin, ultimately degrading fibrin clots.^[^ ^19^ ^]^ Prostaglandin I2 (PGI2) secreted by healthy endothelial cells increases the content of cyclic adenosine monophosphate and activates soluble adenylate cyclase in platelets, thereby inhibiting platelet activation. These two functional molecules have been used to assess the EC monolayer function.^[^ ^18^, ^20^ ^]^ As shown in Figure 3f, the endothelial layer on the A45R1 coating exhibited higher tPA and PGI2 secretion than that on PET and NiTi. Overall, the HUVEC monolayer formed on the A45R1 surface was more compact, stable, and functioned more effectively.

### 2.5 Genes Expression Analysis of HUVECs on A45R1 Coating

**Figure 4:**
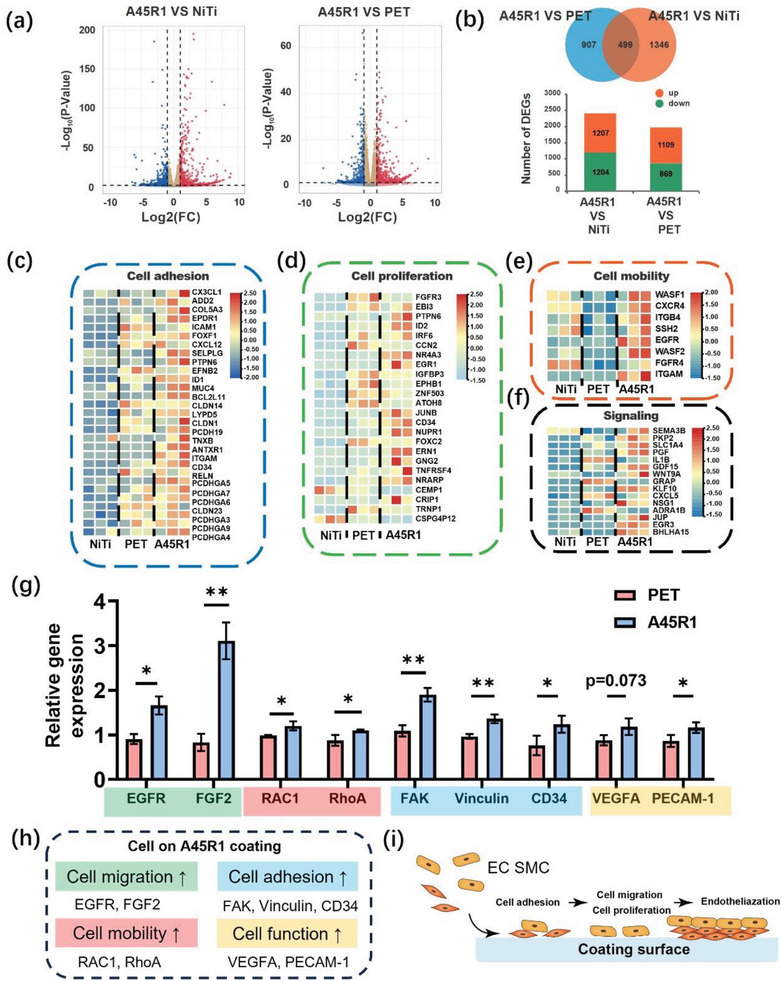
RNA‐seq transcriptome analysis of HUVECs. a) Volcano map of significantly up‐regulated (red) and down‐regulated (blue) genes in different groups. b) Number of DEGs between different groups. Up or down describes the changes in DEGs of the former groups relative to the latter groups. Expression changes in adhesion‐related genes in HUVECs c), proliferation‐related genes d), cell mobility‐related genes e), and signaling‐related genes f). g) RT‐qPCR gene expression analysis of HUVECs cultured on the PET and A45R1 coating. The data were representative of three independent experiments (*n* = 3) and expressed as the mean ± SD. h) Summary of the RT‐qPCR results of the HUVECs on the A45R1 coating. i) Schematics of the relations among cell adhesion, proliferation, migration, and endothelization on the coating surface. Significance determined using unpaired t‐test, ^**^ *p* < 0.01, ^*^ *p* < 0.05.

To conduct a more comprehensive study on the interaction between the A45R1 coating and ECs, we analyzed the gene expression in ECs on the A45R1 surface compared to that on the NiTi alloy and PET surfaces via transcriptome sequencing. **Figure** **4**a shows a volcano plot of the transcriptome sequencing results, demonstrating the differential gene expression between ECs on the A45R1 coating versus those on the NiTi alloy and PET surfaces. Figure 4b displays the number of differentially expressed genes in detail. Compared to the NiTi alloy group, the A45R1 group had 1207 upregulated genes and 1204 downregulated genes, while compared to the PET group, the A45R1 group had 1109 upregulated genes and 869 downregulated genes. We then investigated the functional distribution of the differentially expressed genes. The results of the GO analysis indicated that most of the differentially expressed genes in A45R1 compared to the NiTi alloy and PET were associated with cell binding, cellular components, cell processes, and biological regulation (Figure S3a, Supporting Information). Kyoto Encyclopedia of Genes and Genomes (KEGG) pathway analysis demonstrated that differences in gene expression were concentrated during signal transduction (Figure S3b, Supporting Information). Additionally, we selected representative genes related to cell adhesion, proliferation, motility, and signal transduction to create gene clustering heat maps (Figure 4c–f). These heat maps showed that compared to PET and NiTi alloy, A45R1 significantly improved the expression of genes related to EC adhesion and proliferation, enhanced cell motility, and strengthened signal transduction. We further constructed a protein‐protein interaction network using the STRING database to identify highly concentrated hub node genes and to understand the relationship between these gene expression differences (A45R1 vs PET) (Figure S4, Supporting Information). According to the STRING database, the concentrated genes were mainly related to growth factor activity, positive regulation of angiogenesis, and cellular responses to extracellular stimuli.

We further validated the relationship between endothelial cell gene expression and in vivo endothelialization. We conducted RT‐qPCR for genes that play important roles in cell migration, mobility, adhesion, and function (Figure 4g,h). Cell adhesion to materials is an initiating event in endothelialization.^[^ ^21^ ^]^ The results indicated that on the A45R1 coating, three genes that were highly correlated with cell adhesion (focal adhesion kinase (FAK), vinculin, and CD34) had high expression. This indicates that the coating can effectively promote cell adhesion, which is related to the RGD in the coating. Fibroblast growth factor 2 (FGF2) and Epidermal Growth Factor Receptor (EGFR) control endothelial metabolism and influence EC migration.^[^ ^22^ ^]^ On the A45R1 coating, EGFR and FGF2 showed high expression levels, indicating that the coating promoted EC migration (Figure 4g).^[^ ^23^ ^]^ Rac Family Small GTPase 1 (RAC 1) and Ras Homolog Family Member A (Rho A) are essential Rho GTPases that regulate cell motility.^[^ ^24^ ^]^ The higher expression of RAC 1 and Rho A further confirmed that ECs had a high migration ability on the A45R1 coating. The rapid migration of ECs to the material surface accelerates endothelialization.^[^ ^25^ ^]^ Moreover, genes related to EC function (Vascular Endothelial Growth Factor A, VEGFA) and the EC junction (Platelet And Endothelial Cell Adhesion Molecule 1, PECAM‐1) were expressed at higher levels on the A45R1 coating than on the PET surface, suggesting that the endothelial layer formed on A45R1 exhibited better functionality and tighter junctions. Taking into account the overall gene expression results, the A45R1 coating has the potential to enhance cell adhesion and migration and promote cell functional expression, with the prospect of accelerating the in vivo endothelialization of the device.

### 2.6 Stability of A45R1 Coating on the LAA Occluder

**Figure 5:**
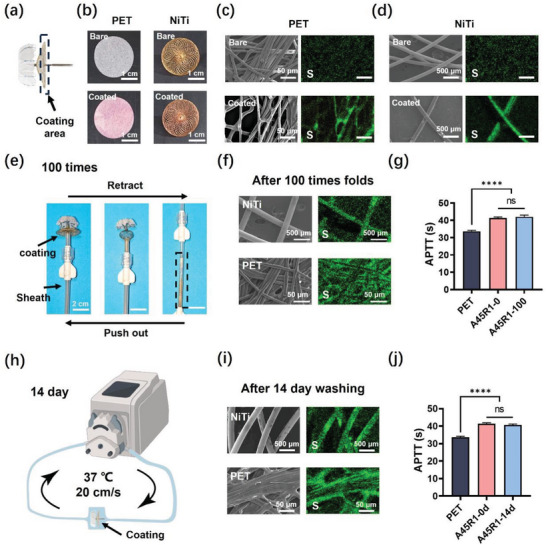
A45R1 coating on the LAA occluder. a) Photograph of the LAA occluder (LAmbre) with A45R1 coating. The arrow indicates the A45R1 coating on the cover disk. The coating was prepared on the whole cover disk. Photographs b) and SEM images and corresponding EDS mapping of the *S* element of the PET fabric c) and the NiTi framework d) before and after A45R1 coating. The coating was dyed using the rhodamine B (b). Scale bars, 1 cm b); 50 µm c); 500 µm d). e) The photographs of the occluder with A45R1 coating undergoing repeated folding processes. Scale bar, 2 cm. f) SEM images (left), and corresponding EDS elemental mapping of S (right) of the A45R1 coating on the NiTi framework and PET fabric after 100 times folds. Scale bar, 500 µm for NiTi and 50 µm for PET fabric. g) APTT of the A45R1 coating after 100 times folds. h) Schematics of the 14‐day washing test for the A45R1 coating on the occluder. Created with BioRender.com. i) SEM images (left) and corresponding EDS elemental mapping of *S* (right) of the A45R1 coating on the NiTi framework and PET fabric after 14 d of washing. Scale bar, 500 µm for NiTi and 50 µm for PET fabric. j) APTT of the A45R1 coating after 14 d of washing. The data were representative of three independent experiments (*n* = 3) and expressed as the mean ± SD. Significance was determined using an unpaired t‐test, ^****^p < 0.0001, ns p > 0.05.

The occluder, owing to its large disc surface and complex structure, emphasizes the importance of coverage and stability of the coating. Further research was conducted to determine the ability and stability of the coating when applied to an occluder. **Figure** **5**a shows an LAA occluder, with the black arrow indicating the cover disk that performed the sealing function and where the coating was applied. Figure 5b provides a macroscopic view of the NiTi framework and PET fabric before and after the A45R1 coating. The staining results for the A45R1 coating (Figure 5b) showed that the A45R1 coating was successfully applied to the entire NiTi framework and PET fabric. We further conducted a SEM and EDS study of the hydrogel coating morphology at the microscale level. From the SEM images, it can be observed that there was no apparent change in the surfaces of the PET fibers and NiTi wires before and after coating (Figure 5c,d). The coating uniformly covered the surface. We further confirmed the coating coverage by elemental mapping of the sulfur element. The uniform coverage of the A45R1 coating was attributed to the excellent wettability of PEI^[^ ^14^ ^]^ and the uniform distribution of the coating precursor liquid achieved via ultrasonic spray coating.

The occluder experienced severe deformation within the sheath during delivery. After implantation, the cover disk of the LAA occluder endures long‐term blood flushing until it is completely covered by the newly formed tissue. Therefore, the stability of the hydrogel coating on the occluder surface is crucial. We simulated the environment during the implantation and post‐implantation of the occluder in vitro. First, we repeated the folding experiments. The occluder needs to be folded and delivered through a sheath tube to the left atrial appendage. The occluder experiences high friction within the sheath tube. In addition, owing to improper delivery positioning, the occluder may need to be retrieved and redeployed (usually not exceeding five redeployments). This process poses a challenge to the surface coating of the occluders. We simulated the process of deploying the occluder 100 times in a 37 °C normal saline environment (Figure 5e) and examined the morphology and anticoagulant properties of the coating. After 100 folding cycles, the A45R1 coating remained intact on the PET fibers and NiTi wires (Figure 5f). The anticoagulant ability of the folded coating was assessed using the APTT test, and the results showed that the coating could still effectively prolong the APTT after 100 folding cycles (Figure 5g). Second, after closure was complete, the occluded surface was subjected to continuous blood flow washing, a process that may also affect the stability of the coating. We studied the coating stability by conducting a 14‐day incubation in a normal saline solution at 37 °C with continuous circulation. After 14‐d incubation, the coating remained intact on the PET fibers and NiTi wires (Figure 5i), and the anticoagulant ability was the same as day 0. We also characterized the adhesion ability of the coating to the substrate using microscratch tests. The black dotted line in Figure S6 (Supporting Information) represents the point at which the probe completely penetrated the coating (the penetration depth did not increase further). The frictional force acting on the probe consistently and uniformly increased with displacement, with no plateau observed, indicating that the coating did not tear or peel during probe sliding. These results demonstrate that the A45R1 coating has excellent toughness and can stably adhere to both the NiTi and PET surfaces. This can be attributed to the presence of the silane coupling agent in the coating. The coating became more stable after 60 °C heating to promote the thermal crosslinking of the silane coupling agent (Figure S5, Supporting Information). The coating also needs to have a mechanical strength matched with the surrounding tissue to avoid damaging the tissue. The dry A45R1 coating had a high modulus of ≈18 MPa, while the wet A45R1 coating had a modulus of ≈700 KPa, which was similar to the soft tissue modulus (Figure S7, Supporting Information). These results demonstrate that the hydrogel coating can be applied evenly to complex structures and exhibits long‐term stability in a physiological environment.

### 2.7 The Canine Model for LAA Occluder

**Figure 6:**
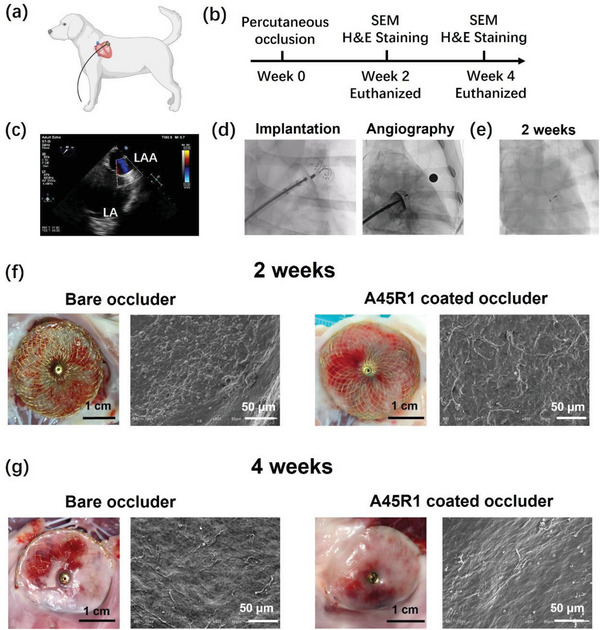
Implantation of the LAA occluder in the canine model. a) Illustration of transcatheter interventional closure of LAA with the bare occluder or the A45R1 coated occluder. b) Illustration of the study design. c) Transthoracic echocardiography (TTE) examination during LAA occlusion. d) The angiography‐guided implantation (left) and residual flow examination (right). e) Angiography image after 2 weeks of implantation. f) Photographs and representative SEM images of the neo‐endocardium coverage on the bare occluder and A45R1 coated occluder at 2 weeks. g) Photographs and representative SEM images of the neo‐endocardium coverage on the bare occluder and A45R1‐coated occluder at 4 weeks. Scale bars, 50 µm in the SEM images.

Encouraged by its promising in vitro performance, we conducted further tests on the A45R1‐coated LAA occluder in a canine model (**Figure** **6**a). The canine LAA anatomy closely resembles that of humans in terms of angulation and morphology^[^ ^26^ ^]^ Moreover, canines have stronger clotting response^[^ ^27^ ^]^ and faster healing properties,^[^ ^26^ ^]^ which make them suitable for testing coating performance in vivo. Four dogs were implanted with bare LAA occluders, whereas four others received A45R1‐coated LAA occluders. The dogs were euthanized at 2 weeks (14 d) and 4 weeks (28 d) post implantation and their LAA walls with occluders were dissected for further evaluation (Figure 6b).

Before implantation, trans‐thoracic echocardiography (TTE) was used to assess the size of the LAA and select the matched occluder size (Figure 6c). The occluders were implanted under the guidance of digital subtraction angiography, and post‐implant angiography was performed to confirm the appropriate closure of the LAA (Figure 6d). Before euthanasia and dissection, TTE was performed to evaluate the device position and any leakage around the device to verify that the occluder was functioning properly (Figure 6e). The overall images in Figure 6d,e confirm the successful implantation and maintenance of function before euthanasia.

After successful implantation, the healing process on the surface of the occluder was analyzed. Macroscopic gross examination of the bare occluders and A45R1‐coated occluders at 2 and 4 weeks (Figure 6f,g, Figures S8, and S9, Supporting Information). Both devices had good contact with the LAA wall, suggesting complete occlusion of the LAA. After 2 weeks, a thin neoendocardium was observed on the A45R1‐coated occluder without thrombus formation (Figure 6f and Figure S8, Supporting Information). By contrast, NiTi wire exposure and thrombus formation were observed on the bare occluder surfaces (Figure 6f, Figure S8, Supporting Information). At 4 weeks, the neoendocardium was observed on both the bare occluder and the A45R1‐coated occluder (Figure 6g, Figure S8, Supporting Information). The A45R1‐coated occluder was completely covered by neoendothelium with several adhering blood cells (Figure 6g, Figure S8, Supporting Information). However, the thrombi were still enwrapped in the neoendocardium, and some NiTi wires were exposed externally (Figure 6g, Figure S8, Supporting Information). Both the photographs and SEM images showed the A45R1‐coated occluders had higher neoendocardium and endothelium coverage than the uncoated device.

**Figure 7:**
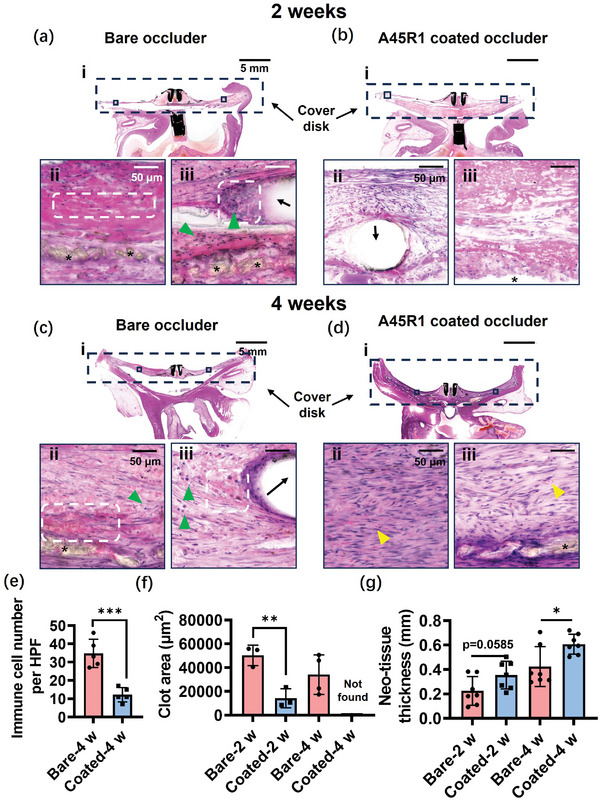
Histological examinations of the LAA occluders implanted in the canine LAA. H&E photograph of the bare occluder a) and A45R1 coated occluder b) at 2 weeks. H&E photograph of bare occluder c) and A45R1 coated occluder d) at 4 weeks. Boxes are in i) and are shown as 40× magnified in ii) and iii). Asterisk (*) indicates PET fabric membranes. The arrow indicates NiTi wire. The white dashed box outlined the thrombus. The green triangles indicated infiltrated leukocytes. The yellow triangles indicated erythrocytes. Scale bars, 5 mm in i) and 50 µm in ii) and iii). Immune cells in the high power field (HPF) e), thrombus area in the neo tissue f), and neo tissue thickness g), on the cover disk were quantified (*n* = 3–7 per group). The data were expressed as the mean ± SD and significance was determined using an unpaired t‐test. *p < 0.05, **p < 0.01, and ***p < 0.001.

To further examine the composition of the surface neo‐tissue and investigate the process of surface endothelialization, histologic examinations were conducted using hematoxylin and eosin (H&E) (**Figure** **7**) and Masson staining (Figure S9, Supporting Information). The results at 2 weeks showed that the A45R1‐coated occluder was covered by thicker early granulation tissue and had less inflammation around the PET fabric or NiTi wires (Figure 7b, Figure S9b, Supporting Information) than the bare LAA occluder surfaces (Figure 7a). However, thrombus and inflammation were observed in the fibrous tissue around the bare PET fabric and NiTi wires (Figure 7a(ii), Figure 7a(iii), Figure S9a, Supporting Information), which slowed down endothelialization. The results obtained after 4 weeks demonstrated significant differences in the neotissue before and after coating. A45R1‐coated occluders were fully covered with thick neotissue (Figure 7d(i)), and the PET fabric and NiTi wires were enveloped by mature fibrous connective tissue with a few erythrocytes inside (Figure 7d, Figure S9d, Supporting Information). The neo‐tissue on the cover disk of the bare occluder was filled with thrombus (Figure 7c(i)), and leukocytes infiltrated around the bare PET fabric and NiTi wire (Figure 7c(ii,iii)). We further quantified the inflammation, thrombus area, and neotissue thickness on the cover disc of the occluder (Figure 7e–g). Compared to the bare PET fabric and NiTi wire, there was a significant reduction in leukocyte infiltration around the PET fabric and NiTi wire after coating for 4 weeks (Figure 7e). After coating, the thrombus area in the neotissue on the cover disk also significantly decreased and quickly disappeared after 4 weeks (Figure 7f). Moreover, the coverage of the newly formed tissue on the cover disk after coating was relatively rapid (Figure 7g).

In general, A45R1‐coated LAA occluders resulted in significantly less thrombus and inflammation and faster neotissue coverage than bare LAA occluders. Based on these findings, we conclude that the A45R1 coating is effective in inhibiting early thrombosis while reducing inflammatory responses, leading to rapid endothelialization of the LAA occluder.

## 3 Conclusion

We developed a functional double‐network polyelectrolyte hydrogel coated on an LAA occluder. Despite the complex structure and multivariate morphology of the LAA occluder, we successfully obtained a firm hydrogel coating on the LAA occluder by optimizing the polyanion and polycation ratios. The in vitro results showed that the hydrogel coating prevented surface thrombus formation; accelerated the adhesion, migration, and proliferation of HUVECs; and improved EC monolayer functions. Notably, a coated LAA occluder promotes neoendocardial formation in a canine model. Therefore, the proposed hydrogel coating is a promising candidate for addressing the complications of interventional LAA occlusion

## 4 Experimental Section

### Materials

The 2‐acrylamido‐2‐methyl‐1‐propanesulfonic acid (AMPS), 4‐methoxyphenol, 2‐hydroxy‐4′‐(2‐hydroxyethoxy)−2‐methylpropiophenone (I2959), polyethylenimine (PEI aqueous solution, 50 w/w%, Mw≈70000), 3‐(trimethoxysilyl) propyl methacrylate (TMSPMA), and rhodamine B were purchased from Aladdin (Shanghai, China). AAc‐GGG‐RGD was purchased from Nanjing Peptide Co. (Nanjing, China). Phosphate‐buffered saline (PBS) was purchased from Sangon Biotech (Shanghai, China). The deionized (DI) water used in all experiments was obtained using a Milli‐Q water purification system (Millipore, Billerica, USA). All reagents were used without further purification. LAA occluders were provided by Lifetech Scientific Co., Ltd. (Shenzhen, China).

### Preparation of PAMPS/PEI and PAMPS/PEI/RGD Hydrogel Coating

First, AMPS, PEI, photoinitiator I2959, and TMSPMA were completely dissolved in water/ethanol (50/50 vol) and degassed before use. For A45 coating precursor solution, 0.09 g AMPS, 0.04576 g PEI solution, 4 µL TMPSMA, and 2 mg photo‐initiator I2959 were dissolved in 2 g 50% water/ethanol solution. For the A35, A40, A50, and A55 coating precursor solutions, the molar ratios of AMPS and PEI were changed (the detailed ratio is presented in Table S1, Supporting Information), and the TMPSMA and I2959 contents remained unchanged. For the A45R coating, AAc‐GGG‐RGD was added according to the AMPS content (for example, in the A45R1 coating solution, the amount of AAc‐GGG‐RGD added was 1% of the molar quantity of AMPS). The substrates or LAA occluders were treated with air plasma (3 min) before the coating procedure. An ultrasonic spray device (frequency: 120KHz) was used to deposit the precursor solution on the substrate. The injection rate of the precursor solution was 50 µL min^−1^. The polymerization was conducted by UV irradiation (120 mW cm^−2^, 365 nm, 300 s), followed by heat condensation (50 °C, 2 h). The hydrogel coating was saturated with deionized water for at least 24 h before testing.

After removing the outer packaging of the occluder, the same steps were performed to treat with air plasma (3 min). The coating precursor solution was then directly deposited on the cover disk surface of the occluder using an ultrasonic spray coating device. The injection rate of the precursor solution was 50 µL min^−1^. Polymerization and heat condensation were performed as described above.

### Characterization of PAMPS/PEI Hydrogel Coating

The coating on the PET sheet and the PET sheet morphology were examined via scanning electron microscopy (SEM, Hitachi S4800), fluorescence microscopy (Nikon DS‐Ri2), and laser scanning confocal microscopy (Olympus FV3000‐OSR). A cross‐sectional element map was acquired using energy‐dispersive X‐ray spectroscopy (EDS, EDAX). X‐ray photoelectron spectroscopy (XPS, K‐alpha) was used to analyze the surface elements. The water contact angle was recorded using a drop‐shaped analyzer (Kruss DSA 100, Germany). Fourier transform infrared attenuated total reflection (FTIR‐ATR) spectra were obtained using an infrared spectrophotometer (Nicolet 6700; USA). Zeta potential was measured using dynamic light scattering (Malvern Instruments).

The swelling ratios of the PAMP/PEI hydrogel coatings were analyzed using a gravimetric method. The samples were immersed in PBS for 24 h to ensure thorough swelling. Subsequently, the swollen coatings were weighed after gently removing the excess solution using filter paper (Ws). Then, the samples were dried in an oven at 70 °C to obtain a constant weight (Wd). The swelling ratios of the samples were calculated using the following equation:

(1) $$\mathrm{Swelling}\;\mathrm{ratio}\;\left(g/g\right)=\frac{Ws-Wd}{Wd}$$

### Blood Compatibility of PAMPS/PEI Hydrogel Coating

Fresh rabbit blood was supplied by the Laboratory Animal Research Center (Zhejiang University) and mixed with 4% sodium citrate for temporary storage at 4 °C.

In the hemolysis test, whole blood was first added to a calcium‐ and magnesium‐free PBS solution, and then red blood cells (RBCs) were isolated from the plasma via centrifugation at 2,000 rpm for 10 min. ≈1 mL of the diluted RBC suspension was added to the samples and incubated at 37 °C for 3 h. PBS was used as the negative control, whereas deionized water was used as the positive control. The suspensions were centrifuged at 8,000 rpm for 3 min, and the absorbance of the released hemoglobin in the suspensions was measured at 540 nm using a UV–Vis spectrometer (Thermo Scientific Multiskan FC, USA).

For the platelet adhesion test, whole blood was centrifuged (1,500 rpm for 15 min) to obtain platelet‐rich plasma (PRP). Samples were incubated with PRP at 37 °C for 2 h and washed thrice with PBS. Samples were fixed with 4% glutaraldehyde solution for 15 min and dehydrated using a series of graded ethanol‐water (v/v %) solutions (20%, 40%, 50%, 60%, 70%, 80%, 90%, and 100%, each for 10 min). Adherent platelets were observed using SEM (Hitachi S4800).

For the dynamic blood incubation tests, the Chandler loop system was used as described in a previous study.^[^ ^28^ ^]^ Briefly, the samples were adhered to the inner wall of the PVC tube. The fresh whole blood containing 4% sodium citrate was mixed with 5% 0.15 mol L^−1^ calcium chloride. The blood was then added to the tubes containing the samples (80% of the loop volume) and closed, forming a round loop. The loops were rotated at 30 rotations min^−1^ at 37 °C for 30 min. Samples were extracted and photographed after cleaning unadhered blood clots.

For the activated partial thromboplastin time (APTT) test, citrated whole blood was centrifuged (3,000 rpm, 15 min) to collect the platelet‐poor plasma (PPP). Samples were incubated with PPP and the APTT reagent at 37 °C in sequence, and the plasma clotting time was recorded.

In the complement activation and contact activation tests, the Human Complement Fragment 3a (C3a) kit and Human Complement Fragment 5a (C5a) kit (MEIMIAN, China) were used to evaluate complement activation (C3a and C5a) of the samples.

### In Vitro HUVECs Behavior on PAMPS/PEI/RGD Hydrogel Coating

HUVECs were purchased from Sciencell Research Laboratories (Danvers, MA, USA). The cells were cultured in an incubator (Thermo, USA) with a humidified atmosphere containing 5% CO_2_ at 37 °C, and the medium for HUVECs was composed of endothelial cell growth medium (Sciencell, USA) supplemented with 5% fetal bovine serum (Sciencell, USA), penicillin (100 units mL^−1^), and streptomycin (0.1 mg mL^−1^) (BDBIO, Hangzhou, China).

For the proliferation and migration test, HUVECs were first stained by CellTracker Red CMTPX Dye. For the proliferation test, HUVECs were seeded onto the surface of the samples and cultured for 4, 24, or 72 h. The cell count at each time point was observed using a fluorescence microscope (ECLIPSE Ti2, Nikon, Japan) and counted by the Cell Count module in Image J (NIH, Bethesda, MD, USA). In the migration test, a 500 µm thick PTFE fence was used to form scratches. The stained HUVECs were seeded onto the samples. After incubation for 12 h, the wounds were removed. The width of the scratch was observed at 0 and 12 h of migration using a fluorescence microscope and evaluated using image J (NIH, Bethesda, MD, USA).

The morphology of the HUVEC monolayer was characterized after 3 d of culture on the substrates. All the samples were gently washed with PBS, fixed with 4% paraformaldehyde, and treated with 0.1% Triton X‐100. Cell staining was performed for nuclei (1:100, DAPI, Sigma‐Aldrich) and CD31 (anti‐CD31, Sigma‐Aldrich). To test the integrity of the HUVEC monolayer, thrombin (3 U mL‐1, T6884, Sigma) was used to treat the monolayer, and the gap between the HUVECs monolayers was observed via fluorescence microscopy.

The function of the HUVEC monolayer was evaluated based on the expression of PGI‐2 and tPA. The culture medium was collected and analyzed using a human PGI‐2 kit and a human TPA ELISA kit (MEIMIAN).

### Transcriptome Analysis of HUVECs

HUVECs were cultured at a density of 50000 cells cm^−2^ on the substrates in three biological replicates per condition. Total RNA was extracted using the TRIzol reagent (Invitrogen, USA). The RNA libraries were sequenced by Major Bio Technology Co., Ltd. (Shanghai, China). Transcripts per million (TPM) were used to measure gene expression by RNA‐seq. Differentially expressed genes (DEGs) were selected with fold change >2 or <0.5 and *p*‐value <0.05 using the R package. Protein–protein associations were analyzed using STRING (https://string‐db.org/). A real‐time polymerase chain reaction (RT‐PCR) assay was employed to analyze the expression of genes involved in EC adhesion, migration, and function, including FAK, CD34, PECAM1, vinculin, RhoA, VEGFA, EGFR, FGF2, and RAC1. Briefly, RNA was extracted from HUVECs cultured on the PET and A45R1 coatings using a TRIzol Plus RNA Purification kit (Invitrogen). After purification, the extracted samples (500 ng) were reverse transcribed into cDNA using SuperScript III First‐Strand Synthesis SuperMix (Invitrogen). The as‐obtained cDNA samples were used as templates for standard PCR analysis using Power SYBR Green PCR Master Mix (Invitrogen). PCR primers were designed to amplify the human genes (Table S2, Supporting Information).

### Stability and Mechanical Properties Tests of PAMPS/PEI/RGD Hydrogel Coating

For the stability of the coating under flow incubation, the NiTi framework and PET fabric with hydrogel coating were cut into a square (0.3 cm width and 2 cm length) and placed in the silicone tube channel with an inner diameter of 0.3 cm. A peristaltic pump was used to connect silicone tubes to form a closed‐loop system. Normal saline was injected into the silicone tube and a flow rate of 84.8 mL min^−1^ (≈20 cm s^−1^) was set to simulate the blood flow rate in the left atrium. The silicone tube was immersed in a 37 °C water bath to simulate physiological temperature. After 14 d of incubation, the occluder was cut off, and the PET fabric and NiTi Framework were removed. The coating on the PET fabric and NiTi Framework was dried and observed using SEM (ZEISS Gemini 300) and surface elemental mapping (EDS, ZEISS Gemini 300).

To ensure the stability of the coating under repeated folding, an LAA occluder (Lifetech LAmbre, 26 mm cover disk, and 20 mm umbrella) with an A45R1 coating on the cover disk was connected to the delivery cable. The cable was slowly retracted and the occluder disk and umbrella were deformed and refolded and finally fully recaptured into the delivery sheath (10F, Lifetech Scientific Co., Ltd). The occluder umbrella and disc were slowly pushed out of the cable (Figure 5h). The fold and reopening of the occluder were performed in 37 °C normal saline. After repeating the fold and reopening process 100 times, the occluder was cut off, and the PET fabric and NiTi Framework were removed. The coating on the PET fabric and NiTi Framework was dried and observed using SEM (ZEISS Gemini 300) and surface elemental mapping (EDS, ZEISS Gemini 300).

The surface stiffness was measured using a nanoindenter (Piuma, tip radius = 26 µm, stiffness 4.26 N m^−1^). The microscratch test was performed using scratch testers (Anton Paar NHT + MCT, USA) that were prepared and equilibrated in water for 24 h before the test.

### Canine Model Implantation and Histopathology Evaluation

All animal experiments were approved by the Animal Care and Use Committee of the Shenzhen Advanced Animal Study Service Center, China. Briefly, eight dogs (25–35 kg) with eight Lifetech LAmbre LAA closure systems (4 coated with A45R1) were used in this study following the guidelines of the Chinese Animal Care and Use Committee standards. The devices were deployed using intracardiac echocardiography (ICE). The device position and stability were confirmed using transthoracic echocardiography (TTE) before releasing each device. After deployment, TTE was performed to evaluate the device position and leakage around the device.

After 2 or 4 weeks, the animals were euthanized, and necropsy was performed. The LAAs with implants were collected from all animals during necropsy. The samples were fixed in 10% formalin for at least 72 h. The LAA device and surrounding LA wall were then dehydrated in a graded series of ethanol solutions. The LAA with the occluder was then divided into two halves, with one half used for histological sections and the other half used for SEM. Detailed information on the animal experiments, histological processing, and histopathological evaluation is provided in the Supporting Information.

### Statistical Analysis

All experimental data were obtained from at least three independent experiments and expressed as the mean ± standard deviation (SD). Statistical significance (*p*) was assessed using SPSS Statistics 26.0 with one‐way analysis of variance (ANOVA), where ^*^ *p* < 0.05 was considered as significant and n.s. was considered as not significant. In the statistical analysis between the two groups, the unpaired Student's t‐test was used with the significance threshold of *p < 0.05, **p ≤ 0.01, and ***p ≤ 0.001.

## Conflict of Interest

The authors declare no conflict of interest.

## Supporting information

Supporting Information

## Data Availability

The data that support the findings of this study are available from the corresponding author upon reasonable request.
