# Supplementary material for: UV‐Triggered Hydrogel Coating of the Double Network Polyelectrolytes for Enhanced Endothelialization
Source: Adv Sci (Weinh). 2024 Mar 28;11(23):2401301. doi: 10.1002/advs.202401301 (PMC11187865; doi:10.1002/advs.202401301)
Supplement: Supplementary file 1 — Supporting Information [file ADVS-11-2401301-s001.pdf]

ADVANCED SCIENCE Open Access

Supporting Information

for *Adv. Sci.*, DOI 10.1002/advs.202401301

UV-Triggered Hydrogel Coating of the Double Network Polyelectrolytes for Enhanced Endothelialization

Xing-wang Wang, Yi-jing Yin, Jing Wang, Hong-mei Yu, Qian Tang, Zhao-yang Chen, Guo-sheng Fu, Ke-feng Ren, Jian Ji* and Lu Yu**

UV-triggered hydrogel coating of the double network polyelectrolytes for enhanced endothelialization

Xing-wang Wang[1,2], *Yi-jing Yin*[2], *Jing Wang*[2], *Hong-mei Yu*[3], *Qian Tang*[1,4], *Chao-yang Chen*[2], *Guo-sheng Fu*[1,4], *Ke-feng Ren*[1,2,4]*, *Jian Ji*[2]*, *Lu Yu*[1,4]*

[1] Key Laboratory of Cardiovascular Intervention and Regenerative Medicine of Zhejiang Province, Department of Cardiology, Sir Run Run Shaw Hospital, Zhejiang University School of Medicine, Hangzhou 310016, China.

[2] MOE Key Laboratory of Macromolecular Synthesis and Functionalization, Department of Polymer Science and Engineering, Zhejiang University, Hangzhou, 310058, China.

[3] Department of Surgery, Sir Run Run Shaw Hospital, Zhejiang University School of Medicine, Hangzhou 310016, China.

[4] Engineering Research Center for Cardiovascular Innovative Devices of Zhejiang Province, Hangzhou 310016, China

* Corresponding to:

renkf@zju.edu.cn (K.-F., Ren)

jijian@zju.edu.cn (J., Ji)

jyulu@zju.edu.cn (L., Yu)

Supplementary methods

left atrial appendage (LAA) occluder implantation in a canine model

Eight dogs (male, 8–10 kg) were implanted with the LAA occluder with or without coating. Specifically, dogs were quarantined in the animal facility to acclimate to the new environment. The quarantine period was 5-7 days. At the end of the quarantine period, a veterinarian assessed their health status, and only dogs deemed healthy in the quarantine inspection were eligible for use in the experiment. Before the surgery, the dogs were weighed, and their health status was recorded.

On the day of the experiment, each dog was intramuscularly injected with xylazine hydrochloride (1-2 mg/kg) for analgesia. The dogs were placed in a supine position on the operating table, intubated, connected to a ventilator, and assisted with ventilation. The tidal volume was set at 10 ml/kg per breath, with a respiratory rate of 10-15 breaths/minute. Anesthesia was maintained by inhaling isoflurane during the surgery. Anesthesia depth was monitored using electrocardiography and the stimulus response of the dog. Before the surgical procedure, heparin sodium injection (150 U/kg) was administered intravenously, and activated coagulation time (ACT) was monitored to ensure that the ACT value was greater than 300s at the beginning of the surgery. Following femoral vein puncture, a 6F arterial sheath was inserted. The transseptal puncture needle was delivered through the atrial septum from the right atrium. After successful puncture, left atrial appendage angiography was performed, measuring the size of the left atrial appendage to select an appropriate size of the occluder. After loading the occluder onto the delivery cable, it was delivered through the delivery sheath to the left atrial appendage. Before the release of the occluder, an assessment was conducted on the stability of the occluder, the residual shunt, and the impact on surrounding tissues. After confirming that the occluder can completely block the left atrial appendage, the delivery cable was rotated to release the occluder. After the surgery, aspirin (100 mg, orally) and clopidogrel (75 mg, orally) were administered daily until the designated endpoint of the experiment during the observation period.

The dogs were euthanized by intravenous injection of saturated potassium

chloride under the deep general anesthesia condition. Gross anatomical examination, histologic evaluation, and scanning electron microscope (SEM) observations were carried out at each time point.

Histology processing for plastics and histopathology evaluation

To dissect the LAA occluder, the left atrium was opened to reveal the cover disk surface of the occluder within the LAA, and photographs were taken. The entire occluder with surrounding LAA was then immersed and fixed in 10% neutral buffer formalin. After more than 72 h of chemical fixation, the LAA with occluder was divided into two halves, with one half used for the histological section and the other half for scanning electron microscopy (SEM). For the SEM samples, after fixation with glutaraldehyde, dehydration was carried out sequentially using ethanol gradients of 80%, 90%, 90%, 95%, and 100%. Critical point drying with carbon dioxide was performed, and the surface of the cover disk was assessed for neointimal coverage using SEM after gold sputtering. For histological section samples, after fixing the tissue samples in formalin, rinsed with running water for about 10 minutes and sequentially dehydrated the samples with a series of ethanol gradients (70%, 80%, 90%, and 100%). The tissues were transparentized with xylene and embedded in methyl methacrylate (MMA) resin. The MMA-embedded samples were sectioned, affixed to slides, and ground to a thickness of approximately 200 μm. The resulting slides were stained with hematoxylin and eosin (H&E) and Masson's trichrome. All slides were scanned using an Olympus VS120 virtual slide system.

For histopathology evaluation, the inflammation, thrombus area, and neo-tissue thickness on the cover disk were evaluated. Screened all cover disk areas on the slide, counted the leukocytes, and measured the thrombus area around the PET fabric and NiTi wire. The leukocyte number in a 40× magnified field of view was quantified (Slide Viewer) to indicate the inflammation. The neo-tissue thickness on the cover disk was measured (Slide Viewer) to indicate the tissue coverage.

Table S1. The EA results of PEI/PAMPS coating.

Sample name	C (%)	S (%)	N (%)	S/N
**A35**	31.03	7.33	9.48	0.773
**A40**	34.39	9.16	8.98	1.02
**A45**	34.46	9.35	9.00	1.04
**A50**	28.51	7.93	7.09	1.12
**A55**	30.85	8.61	7.58	1.14

Table S2 List of RT-qPCR primers

Gene	Genbank Accession	Primer Sequences(5'to3')
Human GAPDH	NM_002046.5	CCATGACAACTTTGGTATCGTGGAA<br>GGCCATCACGCCACAGTTTC
Human CD34	NM_001025109.2	CCTCAGTGTCTACTGCTGGTCT<br>GGAATAGCTCTGGTGGCTTGCA
Human EGFR	U48722.1	AGGGCCTTGCCGCAAAGTGT<br>CCACCGGCAGGATGTGGAGAT
Human FAK	L13616	CTCCTGGTGCAATGGAGCGAGTAT<br>GCAGGTGACTGAGGCGGAATC
Human FGF2	NM_002006.6	CCCAAGCGGCTGTACTGCAA<br>GGTAACGGTTAGCACACACTCCTT
Human PECAM1	NM_000442.4	CACCTCCAGCCAACTTCACCAT<br>CACTGTCCGACTTTGAGGCTATCT
Human RAC1	AF498964.1	CTGCCAATGTTATGGTAGATGGAAA<br>CAGGACTCACAAGGGAAAAGCAA
Human RHOA	NM_001664.2	CCCTCTCCTACCCAGATACCGAT<br>ACGTTGGGACAGAAATGCTTGA
Human VEGFA	NM_001171623.1	GGAGGGCAGAATCATCACGAA<br>GTCCACCAGGGTCTCGATT
Human Vinculin	NM_014000.3	GAGCAAGCACAGCGGTGGATT<br>CGGTCACACTTGGCGAGAAGA

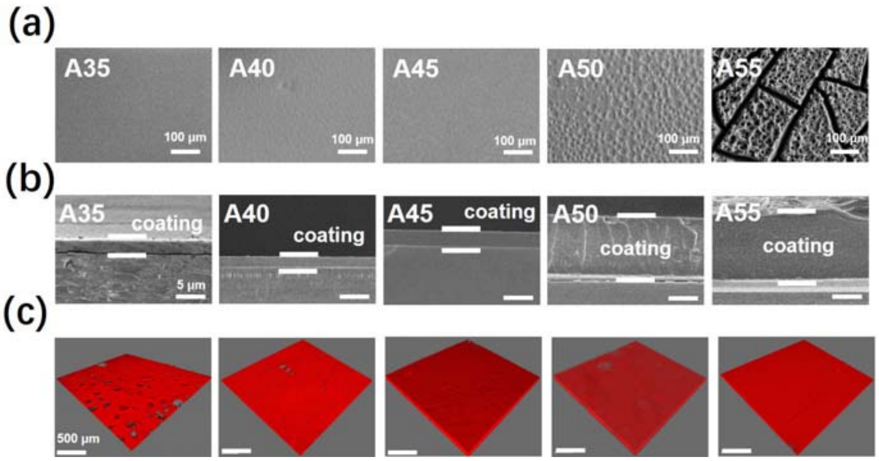

Fig. S1. SEM images (a), cross section image (b), and confocal scanning image (c) of the coating with different mole ratio of AMPS and PEI. Scale bars, (a) 100 μm, (b) 5 μm, (c) 500 μm.

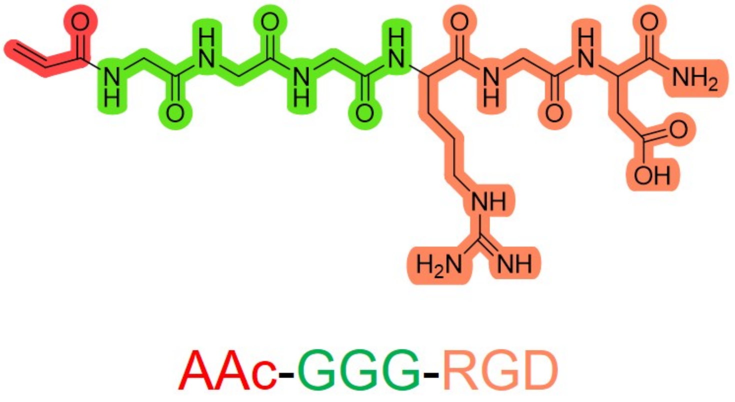

Fig. S2. The chemical structure of the AAc-GGG-RGD peptide

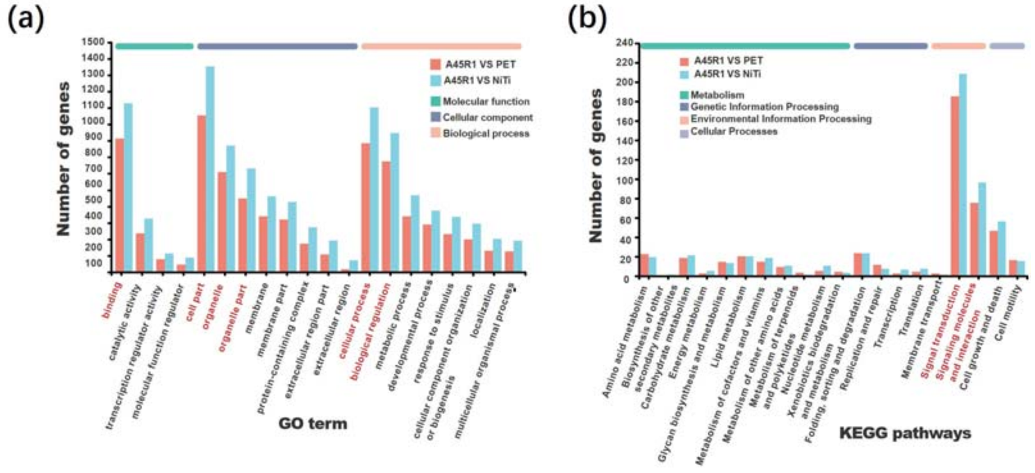

Fig. S3. (a) Significantly enriched GO term (p-value <0.05). (b) Significantly enriched KEGG pathways (p-value <0.05).

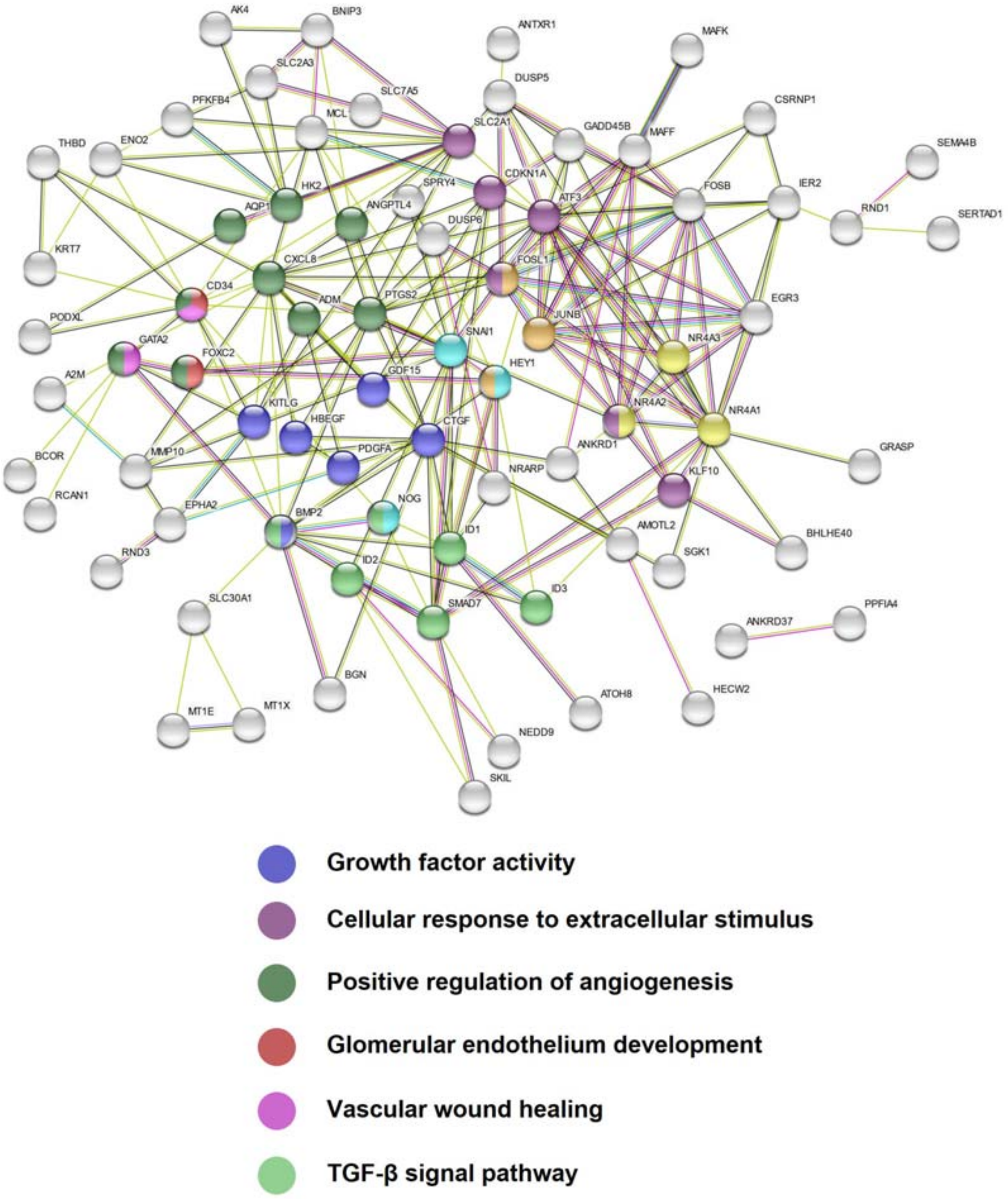

Fig. S4. PPI network analysis of DEGs from HUVECs cultured on A45R1 vs PET.

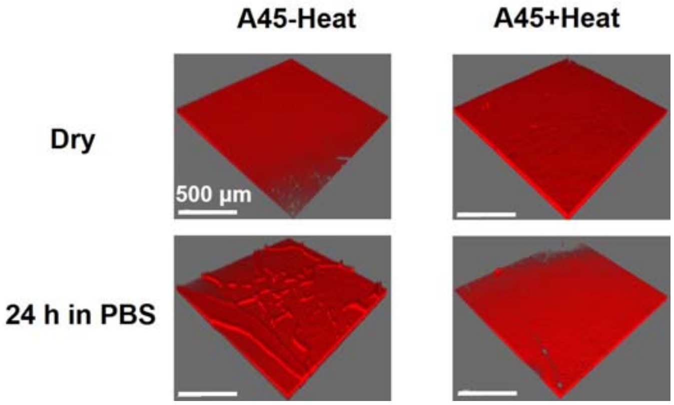

Fig. S5. Effect of the heat treatment on the stability of the A45 coating.

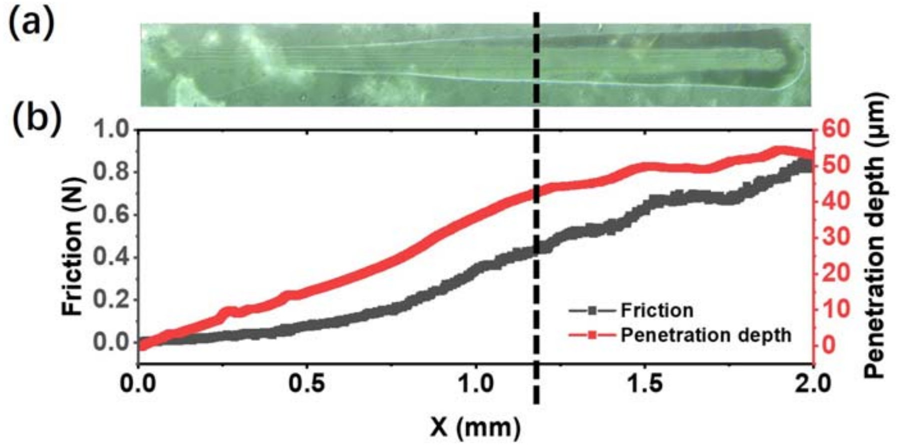

Fig. S6. Micro scratch test of the coating on the PET. (a) Photograph of the micro scratch on the A45R1 coating. (b) Curve of friction (black) and penetration depth (red) variation with displacement.

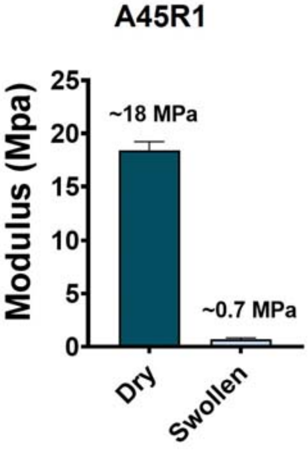

Fig. S7. Stiffness of the A45R1 coating at dry and swollen state.

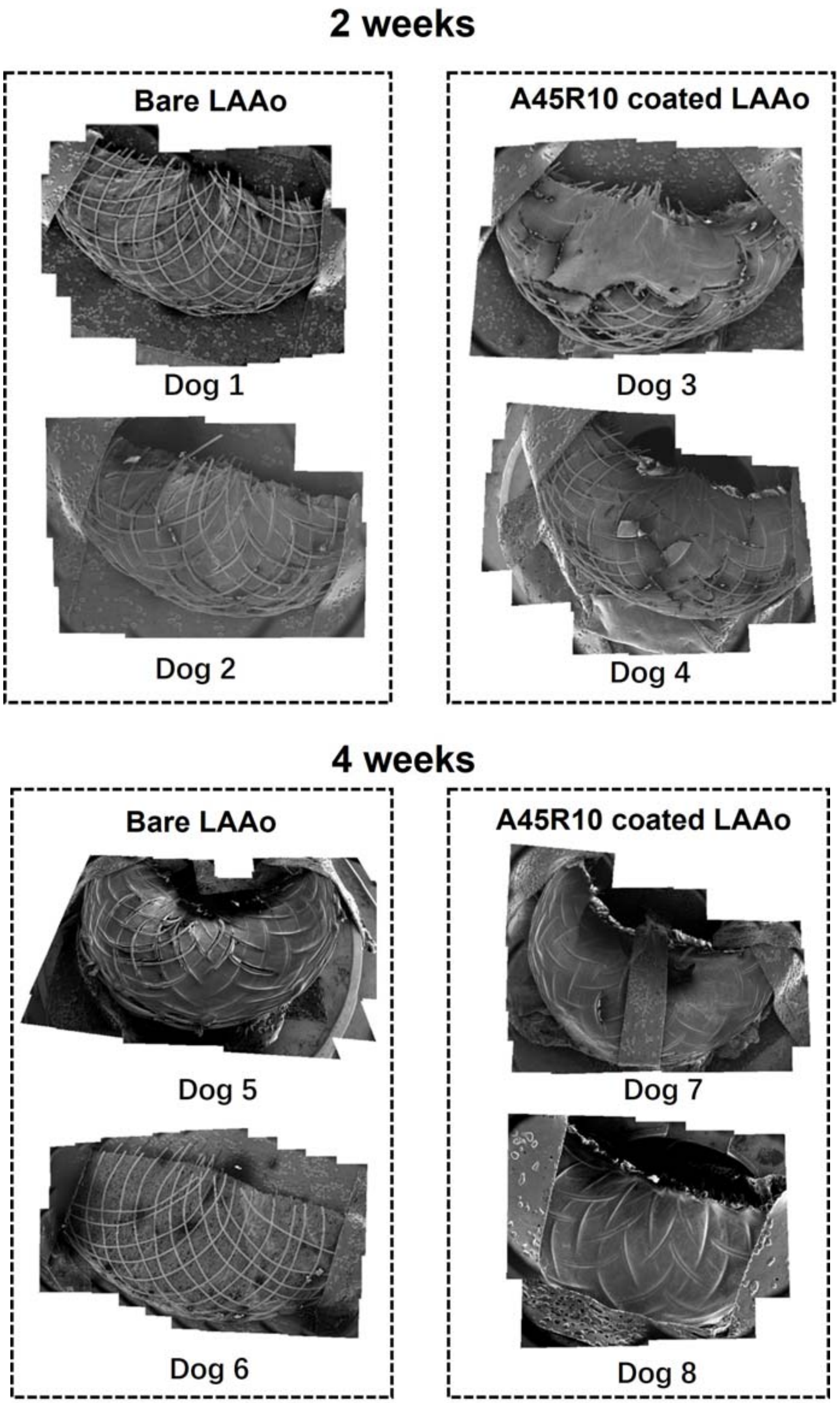

Fig. S8. The large-scale SEM image as the overall view of the occluder disc after 2- and 4-weeks implantation.

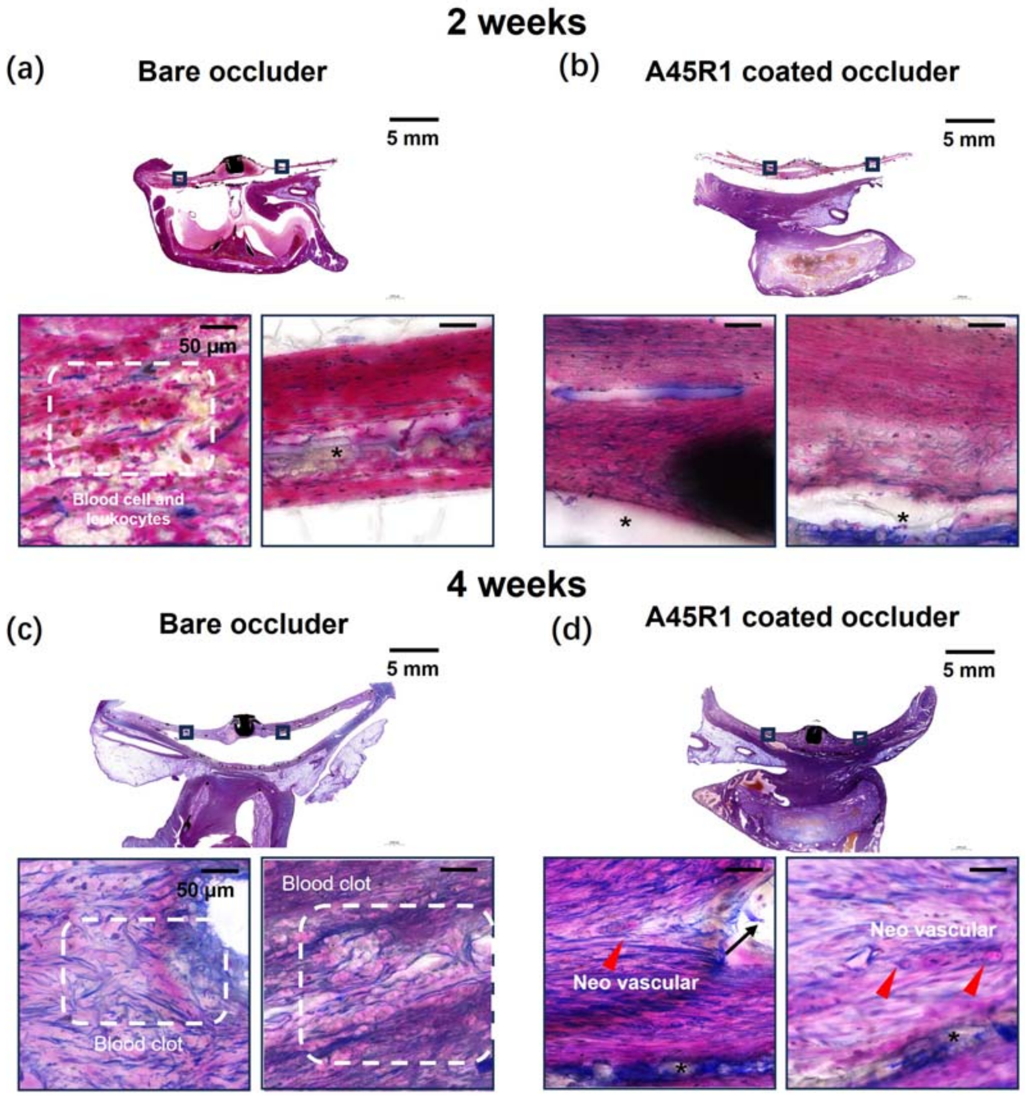

Fig. S9. Histologic examinations of the LAA occluders implanted in the canine LAA. Masson staining results of bare occluder (a) and A45R1 coated occluder (b) at 2 weeks. Masson staining results of bare occluder of bare occluder (c) and A45R1 coated occluder (d) at 4 weeks. Asterisk (*) indicates PET fabric membranes. Arrow indicates NiTi wire. The white dashed box outlined the thrombus. The red triangles indicated neo vascular.
